# Comparative Study of Refraction between Wave Front-Based Refraction and Autorefraction without and with Cycloplegia in Children and Adolescents

**DOI:** 10.3390/children9010088

**Published:** 2022-01-09

**Authors:** Ana M Calvo-Maroto, Sara Llorente-González, Jaione Bezunartea-Bezunartea, Francisco Javier Hurtado-Ceña, Clara Berrozpe-Villabona, Valentina Bilbao-Malavé, David P Piñero, Jesús Barrio-Barrio, Sergio Recalde-Maestre

**Affiliations:** 1 Department of Ophthalmology, Clínica Universidad de Navarra, 28027 Madrid, Spain; sllorente@unav.es; 2 Experimental Ophthalmology Laboratory, Department of Ophthalmology, Clínica Universidad de Navarra, IdiSNA, 31008 Pamplona, Spain; jbezunartea@unav.es (J.B.-B.); srecalde@unav.es (S.R.-M.); 3 Red Temática de Investigación Cooperativa Sanitaria en Enfermedades Oculares, Oftared, ISCIII, 28040 Madrid, Spain; jbarrio@unav.es; 4 Department of Ophthalmology, Clínica Universidad de Navarra, 31008 Pamplona, Spain; 5 Department of Pediatric Ophthalmology, Clínica Rementería, 28010 Madrid, Spain; hurtado@clinicarementeria.es; 6 Department of Preventive Medicine, Public Health and Health Management, Miguel Servet University Hospital, 50009 Zaragoza, Spain; cberrozpe@salud.aragon.es; 7 Department of Ophthalmology, Bellvitge University Hospital, 08907 Barcelona, Spain; valenbilbao@gmail.com; 8 Department of Optics, Pharmacology and Anatomy, University of Alicante, 03690 Alicante, Spain; david.pinyero@gcloud.ua.es

**Keywords:** autorefraction, cycloplegia, children, accommodation, refractive errors

## Abstract

The main aim of this study was to compare refraction measurements with and without cycloplegia from two refractors devices, (TRK-2P autorefractometer (TRK-2P) and wavefront-based refraction Visionix 130 (VX130)) in children and adolescents. This descriptive observational study included 20 myopic eyes and 40 hyperopic eyes measured in two different Spanish hospitals. Cycloplegia was carried out by three drops of cyclopentolate hydrochloride 1% (Colircusí cycloplegic, Alcon Healthcare S.A., Barcelona). The mean age of the myopia group was 12.40 ± 3.48 years; for the hyperopia group, the mean age was 7.37 ± 2.47 years. In the myopia group, autorefraction and wavefront-based refraction did not show clinically significant differences in any components between with and without cycloplegia. The hyperopia group showed statistical and clinically significant differences in sphere and SE components between relaxed and non-relaxed states of accommodation, although the cylindrical components were not clinically different. In this study, we considered a value of ≥0.50D as a clinically significant difference in refraction. Therefore, both devices were capable of obtaining accurate refractions without cyclopegia in myopia children, although they did not avoid instrument myopia and accommodation involved in hyperopia children. Moreover, both refractometers could be useful for astigmatism monitoring in children without the need for cycloplegic drops.

## 1. Introduction

Refractive errors, such as myopia, hyperopia, and astigmatism, affect a large proportion of the population worldwide. In particular, myopia prevalence has increased dramatically in the last decades; a meta-analysis suggested that close to half of the world’s population may develop myopia by the year 2050, with as much as 10% highly myopic [1]. According to this, nearly 5 billion people will be myopic, and 1 billion people will have high myopia [1]. The European Eye Epidemiology (E3) Consortium concluded that estimates of refractive error prevalence in Europe are 30.6% for myopia, 2.7% for high myopia, 25.2% for hyperopia, and 23.9% for astigmatism [2,3]. Age-specific estimates showed a high prevalence of myopia in younger participants (47.2%), specifically in those with ages between 25 and 29 years old [2,3].

In children, these refractive errors may not be easily detected due to children often not complaining of visual defects or not being aware of the need for correction. The consequences of uncorrected refractive error have an important effect over educational and psychosocial development [4,5]. Moreover, high myopia increases the risk for developing several pathological ocular conditions, such as cataract, glaucoma, retinal detachment, and myopic maculopathy [6,7], that may cause an irreversible loss of vision later in life [8].

Subjective manifest refraction is considered the gold standard for refraction and spectacles refraction [9], although it is a time-consuming procedure in clinical practice. Retinoscopy and objective autorefraction are considered essential to determine and correct refractive errors in children, and they are often used as the starting point for the subjective refraction [10]. Several authors have demonstrated that autorefraction provides a repeatable and accurate measurement of refractive errors [11,12]. However, several studies have revealed that the use of these devices in children without cycloplegia could underestimate hyperopia and overestimate myopia [13,14,15,16], due to the accommodation of children, which interferes with the diagnostic precision of the latent refractive errors [16]. Therefore, child and adolescent ophthalmology examination requires cycloplegic refraction to determine an accurate refraction [13,17]. 

Due to difference in technology of the different devices to measure refractive errors, the increase in the prevalence refractive errors, and the activities with high demands on near vision [18], the aim of this pilot study was to compare refractive error measures without and with cycloplegia in a TRK-2P autorefractometer (TRK-2P) and wavefront-based refraction obtained with a VX130 in children and adolescents (age up to 18 years old) and to examine these refractive measurements between both devices. This study also aimed to assess the agreement of refraction measures between both methods and how the capacity of accommodation may affect the refraction measurements in both devices.

## 2. Materials and Methods

### 2.1. Subjects

We have studied 60 eyes from 60 patients. All subjects were recruited from the Department of Ophthalmology of Clínica Universidad de Navarra and Clínica Rementería, Madrid (Spain). They were selected from consecutive cases among the clinic population that met our inclusion criteria. One eye per patient was randomly chosen for this study according to a random number sequence (0 and 1) to avoid bias related to the use of both eyes of the same patient. All procedures carried out in this study conformed to the guidelines of the Declaration of Helsinki. The institutional Review Board and the Ethics Committee of Clínica Universidad de Navarra and Clínica Rementería (Spain) approved the protocols used in this study. All patients and their parents were fully informed of the purpose and procedures, and informed consent was obtained from each parent.

Inclusion criteria were subjects with age below 18 years old and best-corrected visual acuity (BCVA) of 20/25 or better. Exclusion criteria were previous corneal and/or ocular surgery, previous corneal or ocular disease, clinical corneal changes, and the use of contact lenses. Subjects were excluded if they did not cooperate for autorefraction examination with both devices used. All subjects had no other ocular and systemic complications.

All subjects underwent a comprehensive ophthalmic evaluation including autorefraction by TRK-2P (Topcon Corporation, Tokyo, Japan) and VX130 (Visionix Luneau, Chartres, France), uncorrected visual acuity (UCVA), subjective refraction, BCVA, slit-lamp biomicroscopy, and ocular fundus examination. After this, three drops of cyclopentolate hydrochloride 1% (Colircusí cycloplegic, Alcon Healthcare S.A., Barcelona, Spain) were instilled every 10 min in both eyes. Autorefraction, manifest refraction, and BCVA were measured 60 min after the first instillation. Cycloplegic autorefraction was carried out with both TRK-2P and VX130 devices.

Patients were divided by refractive errors (spherical equivalent) under cycloplegia measured with TRK-2P into myopia ≤−0.50D, hyperopia ≥+0.50D, and emmetropia between −0.50 and +0.50D [13,19]. In the hyperopic group, the maximum cylinder was considered so that spherocylindrical refraction transformation into vectorial notation did not cause a change in the refraction group.

### 2.2. Procedures

The TRK-2P (Topcon Corporation, Tokyo, Japan) is an automated measurement system that combines autorefractometer, keratometer, non-contact tonometer, and pachymeter. For refraction measurements, this instrument projects a near-infrared light to the retina, and the reflected image is received by a charge-coupled device (CCD) camera; the spherical refractive power, cylindrical refractive power, and the axis of astigmatism are determined through proprietary algorithms. Three consecutive refraction measurements were taken, and the internal software provided an average of these measurements that was used in statistical analysis.

The VX130 (Visionix Luneau Technologies, Chartres, France) is a multi-diagnostic platform that combines refraction (Hartmann–Shack-based autorefractometer), a Placido disk corneal topographer, a Scheimpflug imaging-based system, a Hartmann–Shack wavefront aberrometer, and an air tonometer. The VX130 uses a short flash of blue LED light of wavelength 850 nm for refractive error measurements and with a constant power of 50 μm. The Placido disk system projects 24 rings on the corneal surface, measuring more than 100,000 points. This information is used to provide all corneal topographic information. The Scheimpflug imaging-based system uses monochromatic blue light of 450 nm to obtain pachymetry and iridocorneal angle measurements. The Hartmann–Shack aberrometer measures 1500 points in 0.2 s in an area ranging from 2.0 to 7.0 mm^2^ of diameter.

The ocular refraction was measured over the complete pupil size, detected automatically by the devices. However, to determine autorefraction readings, the internal software fits the evaluated pupil size to a diameter of 3mm. Therefore, all measurements were taken for a 3mm pupil size, with a distance vertex of 12 mm in both devices.

### 2.3. Spherocylindrical Refraction

The spherocylindrical refractions were converted to vectorial notation using the power vector method as described by Thibos et al. [20]. Thus, sphere, cylinder, and axis components were transformed into M, J_0_, and J_45_ coefficients, where M was the spherical equivalent (SE) (M = sphere + [cylinder/2]), J_0_ was the Jackson cross-cylinder at axis 0° (J_0_ = −[cylinder/2] cos [2 **×** axis]), and J_45_ was the Jackson cross-cylinder al axis 45° ( J_45_ = −[cylinder/2] sin [2 **×** axis]). 

### 2.4. Statistical Analysis

The normality of data samples was evaluated using the Kolmogorov–Smirnov test. Differences between automated refraction with and without cycloplegia and their degree of agreement were assessed using Bland–Altman analysis [21]. Bland–Altman analysis included the calculation of the mean difference between conditions, the standard deviation (SD), and the 95% limits of agreements (LoA = mean difference ± 1.96 × standard deviation of the difference). Moreover, 95% confidence intervals (CI) for the upper and lower limits of agreement were calculated (myopia: limit of agreement ± (2.09 × standard error); hyperopia: limit of agreement ± (2.02 × standard error) [22].

For paired data, Student *t*-test was used to assess the difference between variables with and without cycloplegia. 

In all tests, differences were considered statistically significant when the *p* value was less than 0.05. Data analysis was performed using SPSS for Windows (version 19, SPSS, Inc., Chicago, IL, USA) and Graphpad 8.0 (GraphPad Software, Inc., San Diego, CA, USA).

## 3. Results

A total of 60 eyes from 60 normal subjects were enrolled in this study. The mean age was 8.97 ± 3.67 years (range 3–18 years). A total of 20 myopic eyes and 40 hyperopic eyes were included in this study. The mean age of the myopia group was 12.40 ± 3.48 years (range 4–18 years); for the hyperopia group, the mean age was 7.37 ± 2.47 years (range 3–15 years). The age of the hyperopia group was statistically different from the myopia group (*p* < 0.001).

### 3.1. Comparison of Refractive Components with and without Cycloplegia

Table 1 reports the mean and SD of sphere, cylinder, SE, and J_0_ and J_45_ cylinder components obtained by TRK-2P in both refractive groups. In the myopia group, sphere and SE showed a non-statistically significant increase of 0.16D and 0.14D, respectively, after cycloplegia (*p* = 0.137, *p* = 0.209, respectively). For the cylinder component and J_0_ and J_45_ cylinder coefficients, a decrease in three variables was observed (−0.05, −0.03D and −0.01D, respectively), although these difference were non-statistically significant in any refractive components (*p* = 0.137, *p* = 0.613, *p* = 0.209, *p* = 0.098, *p* = 0.541; respectively). 

However, in the hyperopia group, sphere and SE report a statistically significant increase of 1.57D and 1.52D after cycloplegia (both *p* < 0.001). For astigmatism components, these differences were also statistically significant (J_0_: 0.06D *p* = 0.002; J_45_: 0.24D *p* = 0.005), except for the cylinder component (*p* = 0.074).

With the VX130, sphere and SE showed a significant difference of 0.18D and 0.17D after cycloplegia in the myopia group (*p* = 0.019, *p* = 0.012, respectively). The cylinder component had a decrease of 0.03, although this difference was not statically different. The cylinder coefficients did not undergo any change after cycloplegia (Table 2). 

In the hyperopia group, a significant increase of 1.12D and 1.17D was found in sphere and SE after cycloplegia (both *p* < 0.001). The J_0_ cylinder component showed a slight but not statistically significant (*p* = 0.407) decrease of 0.02D, although the cylinder showed a decrease of 0.09D and the J_45_ coefficient reported a significant increase of 0.05D after cycloplegia (*p* = 0.014, *p* = 0.009, respectively). 

### 3.2. Comparison between Instruments without Cycloplegia

**Figure 1:**
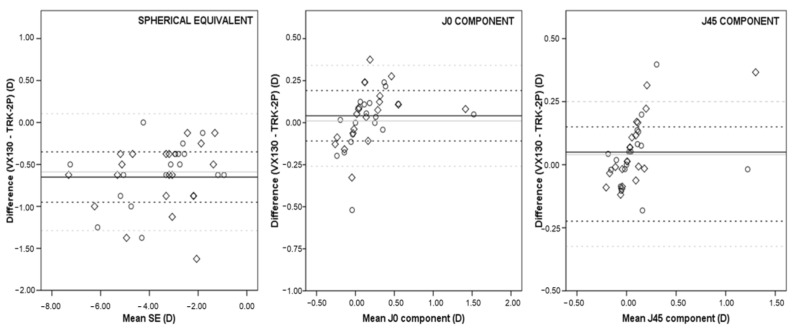
Differences in spherical equivalent (**left**), J_0_ component (**center**), and J_45_ component (**right**) between Visionix 130 system (VX130) and TRK-2P autorefractometer (TRK-2P) in myopia group without cycloplegia (rhombus; mean bias represented by a solid black line and the 95% limits of agreement by dashed black lines) and with cycloplegia (circle; mean bias represented by a solid grey line and the 95% limits of agreement by dashed grey lines).

Bland–Altman plots of SE, J_0_, and J_45_ between both devices for the myopia group are shown in Figure 1. The mean difference for SE showed a clinically significant difference of −0.651 ± 0.407D between both devices, and 95% LoA of 0.147 to −1.449D (or ± 0.798D), respectively. In the J_0_ coefficient, the mean difference was 0.014 ± 0.166D, with 95% LoA of 0.367 to −0.284D (or ± 0.326D) and a mean difference of 0.053 ± 0.141D and 95% LoA 0.331 to −0.224D (or ± 0.277D) for the J_45_ coefficient. 

For SE, the 95% CI for the upper limit of agreement is 0.342 to −0.048D and for the lower limit of agreement is −1.254 to −1.645D. Related to astigmatism components, the 95% CI for the upper limit of agreement is 0.446 to 0.287D and for the lower limit is −0.204 to −0.364D in the J_0_ coefficient. For the J_45_ component, the 95% CI for the upper limit of agreement is 0.398 to 0.262D, and for the lower limit, it is −0.156 to −0.292D.

**Figure 2:**
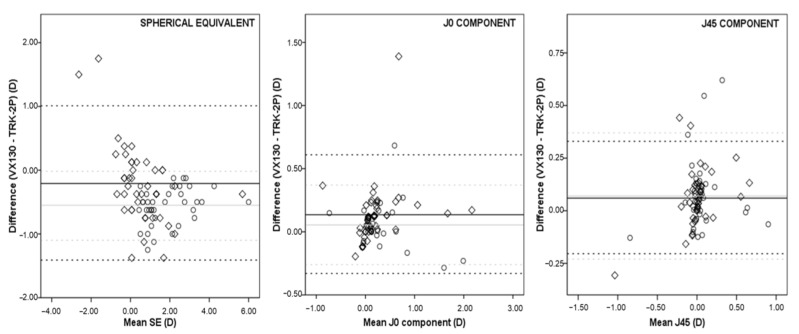
Differences in spherical equivalent (**left**), J_0_ component (**center**), and J_45_ component (**right**) between VX130 and TRK-2P in hyperopia group without cycloplegia (rhombus; mean bias represented by a solid black line and the 95% limits of agreement by dashed black lines) and with cycloplegia (circle; mean bias represented by a solid grey line and the 95% limits of agreement by dashed grey lines).

In the hyperopia group, the mean difference and 95% LoA for SE were −0.209 ± 0.626D and 1.018 to −1.437D (or ± 1.227D), respectively. For the J_0_ coefficient, the mean difference was 0.135 ± 0.242D and 95% LoA were from 0.611 to −0.340D (or ± 0.475D). The mean difference and 95% LoA for J_45_ were 0.063 ± 0.136D and 0.331 to −0.204D (or ± 0.267D), respectively (Figure 2).

In this subgroup, the 95% CI for the upper limit of agreement is 1.217 to 0.817D and for the lower limit of agreement is −1.236 to −1.636D in SE. For the J_0_ coefficient, the 95% CI for the upper limit of agreement is 0.689 to 0.534D, and for the lower limit, it is −0.262 to −0.417D; for the J_45_ component, the 95%CI is 0.374 to 0.287D and −0.161 to −0.247D for the upper and lower limit of agreement, respectively.

### 3.3. Comparison between Instruments with Cycloplegia

In the myopia group, Bland–Altman plots of SE showed at SE had a clinically significant difference of −0.592 ± 0.355D and the 95% LoA were from 0.105 to −1.289D. For the J_0_ coefficient, the mean difference and 95% LoA were 0.010 ± 0.180D, and from 0.362 to −0.342D, respectively. The J_45_ component reports a mean difference of 0.044 ± 0.123D and 95% LoA from 0.286 to −0.197D.

For SE, the 95% CI for the upper limit of agreement is from 0.275 to −0.065D, and for the lower limit of agreement, it is −1.119 to −1.459D. For the J_0_ coefficient, the 95% CI for the upper limit of agreement is from 0.448 to 0.276D and the lower limit is −0.256 to −0.429D, and for the J_45_ component, the 95% CI for the upper limit of agreement is from 0.344 to 0.227D and for the lower limit of agreement is −0.138 to −0.256D.

In the hyperopia group, Bland–Altman plots of SE showed a mean difference of −0.560 ± 0.276D, and the 95% LoA were from −0.019 to −1.100D. For the J_0_ component, the mean difference and 95% LoA were 0.054 ± 0.162D, and from 0.972 to −0.263D, respectively. The J_45_ coefficient showed a mean difference of 0.072 ± 0.154D and 95% LoA from 0.374 to −0.231D.

For SE, the 95% CI for the upper limit of agreement is 0.069 to −0.106D, and for the lower limit of agreement, it is −1.012 to −1.188D. For the J_0_ coefficient, the 95% CI for the upper limit of agreement is 0.424 to 0.320D, and for the lower limit, it is −0.211 to −0.315D; for the J_45_ component, the 95% CI is 0.423 to 0.325D and −0.182 to −0.280D for the upper and lower limit of agreement, respectively.

## 4. Discussion

The determination of refractive errors is essential for cataract surgery [23], refractive surgery [24], pediatric ophthalmology [10], and others. In clinical practice, refractive errors can be obtained by retinoscopy, subjective refraction, and automatic devices, such as autorefractometers and multi-diagnostic platforms. However, in children and adolescents, an ocular examination with cycloplegia is necessary for an accurate refraction [19,25], since uncorrected refractive errors in children, particularly in hyperopia, could promote the development of amblyopia or visual acuity impairment [26,27]. 

The validity and repeatability of the use of autorefraction to determine refractive errors have been widely studied in children [13,28,29]. So, the aim of the current study was to compare refractive error readings in children and adolescents with and without cycloplegia from TRK-2P and the VX130. To our knowledge, this is the first study evaluating the clinical application of both automatic devices in a pediatric and adolescent population (3–18 years old).

In this study, hyperopic patients were younger than myopic subjects (*p* < 0.001). This can be due to normal development of the eye that is oriented towards emmetropia or lower hyperopia (emmetropization process) during infancy and childhood. At the age of 6 years, the mean refractive value is around +0.75D, and after this age, the refractive error is a combination of emmetropization process and visual experience of children [30,31]. 

We considered a value of ≥0.50D clinically significant as this is considered as the minimum dioptric range that can be tolerated in retinal image quality and whose change is not appreciable in terms of a lack of sharpness in focus. This dioptric range is called depth of focus, and it determines the accuracy of refraction [32].

### 4.1. Comparison of Refractive Components with and without Cycloplegia

In relation to TRK-2P, our results showed a non-statistically significant positive shift of sphere and SE of myopia under cycloplegia (0.16 *p* = 0.137; 0.14D *p* = 0.209, respectively), although these differences were not clinically significant. A clinically and statistically significant increase in sphere and SE was observed in the hyperopia group after cycloplegia (1.57D and 1.52D, both *p* < 0.001).

VX 130 also showed a positive change of sphere and SE in myopia group under cycloplegia (0.18D *p* = 0.019, 0.17D *p* = 0.012, respectively), but it was not clinically significant. However, the hyperopia group underwent a statistically significant increase in sphere and SE (1.12D and 1.17D, both *p* < 0.001). 

Therefore, we observed that both devices are capable of minimizing/paralyzing accommodation in the myopia group, but not in the hyperopia children. It is well known that the instrument myopia is large in hyperopia where accommodation tends to be involved. These results support the previous finding that myopic eyes show less accommodation for near targets, therefore minimizing the difference between cycloplegic and noncycloplegic refractions [33]. 

Both devices could be useful for astigmatic monitoring in children without the need for cycloplegic drops since differences were not found to be clinically significant between both conditions and were consistent with previous studies in children [10,13]. 

Moreover, VX 130 provided more negative measures in relaxed and non-relaxed states of accommodation in both groups, and there was a clinically significant difference in SE in the myopia group between both devices before and after cycloplegia (0.65D and 0.59D, respectively), which, for the hyperopia group, were only observed under cycloplegia (0.56D). 

These differences might be explained by several factors: First, accommodation shifts for closeness of instrument (instrumental myopia). Some studies have found more myopic refractions by different aberrometers [34]. Second, repeatability of aberrometric readings: some authors have demonstrated short-term variations related to accommodation and small fixational eye movements [35]. Third, TRK-2P was used before VX130 for clinical protocol to obtain refractive readings; thus, the use of TRK-2P could develop a slight accommodation that was reflected on the VX130 system refraction readings. 

Our results agree with previous studies that suggested that autorefraction without cycloplegia does not paralyze accommodation, showing an over-negative refraction measurement [10,11,13]. Sankaridurg et al. compared autorefraction by KR-8900 autorefractor with and without cycloplegia in children (4–15 years), and they showed an SE mean difference between noncycloplegic and cycloplegic refraction of −0.63D (noncycloplegic error was more myopic), −0.65D for sphere, and −0.04D for cylinder; all were statistically significant. They concluded that these differences were explained by an accommodative response to proximal cues during noncycloplegia [36]. Rotsos et al. compared refraction measurements from RMA-3000 autorefractometry with retinoscopy after cycloplegia in children (3–15 years). They found that SE was significantly more positive after cycloplegia in an autorefractomer (0.75D) in myopic children. For hyperopic children, the sphere was significantly higher under cycloplegia (0.75D) [15]. Hiraoka et al. measured refractions in hyperopic children (3–12 years), and they found a significant increase of 1.18D for SE.

However, Hashemi et al. compared the sphere and cylinder refraction between retinoscopy and autorefraction with and without cycloplegia in children. They observed that autorefraction tended to over plus hyperopics and under minus myopic children compared with retinoscopy; these differences were statistically significant but not clinically significant. They concluded that the autorefractor can be interchanged in refractions measurements under cycloplegia [37].

Other studies have used different models or autorefractor, as handheld Retinomax of photorefraction showed the same tendency as that of the autorefraction. Rajavy et al. reported that photorefraction without cycloplegia in children (age 7–12 years) showed a greater myopic shift of SE of 1.21D in hyperopia [38]. 

Yassa et Ünlü reported an SE increase of 1.16D in children after cycloplegia. For the spherical component, this increase was 1.17D under cycloplegia. For the cylinder component, there was no difference in photorefraction with Plusoptix A09 [39]. Tuncer et al. compared handheld Retinomax readings in children. They found that the sphere and SE underwent an increase of 0.63D and 0.71D, respectively, under cycloplegia. For the cylinder, an increase of 0.15D was found after cycloplegia [12]. Saini et al. also reported an increase of 0.94D and 0.93D in the sphere and SE, respectively, in children (3–7 years). They found a cylinder difference of 0.02D after cycloplegia with an autorefractor [40]. Payerols et al. found an SE increase of 1.02D after cycloplegia in a Nidek ARK-530A autorefractor in children older than 3 years, and of 3.16D under cycloplegia in a portable Retinomax autorefrator in children younger than 3 years. For the cylinder, an increase of 0.25D was observed in Nidek ARK-530 A and of 0.27D for Retinomax under cycloplegia [41].

A clinically significant underestimation of readings without cycloplegia was observed in the hyperopia group compared with the cycloplegia condition in both devices. A myopic overcorrection was observed, although it was not clinically significant with both devices. So, VX130 and TRK-2P could be able to paralyze instrument myopia and child accommodation in myopia but not in hyperopia. It is well known that the instrument myopia is large in hyperopia where accommodation tends to be involved. These differences without and with cycloplegia between both devices were similar to those reported in previous studies [10,11,13]. Both devices could be useful for astigmatic monitoring in children without the need for cycloplegic drops since the differences found were not clinically significant between both conditions and were consistent with previous studies in children [10,13]. 

### 4.2. Agreement between Instruments without Cycloplegia

Regarding the SE and cylinder coefficients, both devices cannot be considered interchangeable for SE measurements as the differences obtained in this agreement were clinically significant. 

### 4.3. Agreement between Instruments with Cycloplegia

A decreased in SD and the Bland–Altman plots range was observed after cycloplegia compared to those results obtained without cycloplegia in both devices (Figure 1 and Figure 2). These results support those obtained by Suryakumar et al., and they suggest that these differences might be due to changes in accommodative behavior in children [28].

However, these decreases were not enough to be able consider both devices as interchangeable in refractive components measurements under cycloplegia. 

This pilot study has several limitations: (I) subgroups of patients did not have a similar sample size, (II) no sample size calculation was carried out, and (III) a comparison with the gold standard cycloplegic retinoscopy was not performed. However, previous studies have showed good agreement between autorefractometer and retinoscopy under cycloplegia in children [42,43,44]. Prabakaran et al. compared table-mounted, hand-held autorefractors with retinoscopy under cycloplegia in children (mean age 52.3 months) and showed that differences between table-mounted autorefraction and retinoscopy were not statistically significant, and they concluded that refractive errors were similar between both devices [42]. Guhan et al. observed that differences between refractive components were not statistically significant between autorefraction under cycloplegia and retinoscopy, and they concluded that the autorefractor can be used reliably in children older than 6 years, and in children less than 6 years, refractions should be corroborated with retinoscopy [44]. A review of the American Academy of Ophthalmology (AAO) evaluated the accuracy of autorefraction compared cycloplegic retinoscopy in children and concluded the use of cycloplegic autorefraction is appropriate in pediatric population, and cycloplegic autorefraction can be valuable by cycloplegic retinoscopy in individual cases when results were not consistent with the expected results [45]. Another study compared wavefront-based refraction between autorefraction, and it was observed that differences in refractive errors were not clinically significant [46]. To our knowledge, there are no other comparative studies between wavefront-based refraction and retinoscopy under cycloplegia.

Therefore, more studies are necessary with a cycloplegic retinoscopy comparison and devices studied, and with a similar sample size of refractions groups with higher myopia/hyperopia subgroups due to ocular consequences in children. 

## 5. Conclusions

In conclusion, TRK-2P and VX130 showed values minus negative of myopia under cycloplegia, but these differences were not clinically significant. For hyperopia, both devices exhibited refractions readings minus positive of hyperopia before cycloplegia. In spite of being based on different technologies, we found a slight overestimation of myopia and underestimation of hyperopia in both devices, and they can paralyze the child accommodation and instrument myopia. Moreover, both devices could be useful for astigmatism monitoring in children without the need for cycloplegic drops. However, the wavefront-based refraction (VX130) system provides measures that are more negative than those obtained by autorefraction (TRK-2P) without and with cycloplegia in children and adolescents.

## Figures and Tables

**Table 1:** Refractive components of both groups measured by TRK-2P with and without cycloplegia.

Refractive Error	Parameters (Mean ± SD)	Pre-Cycloplegia	Post-Cycloplegia	*p*
Myopia (*n* = 20)	Sphere (D)	−3.04 ± 1.71	−2.88 ± 1.66	0.137
	Cylinder (D)	−0.55 ± 0.74	−0.60 ± 0.81	0.163
	SE (D)	−3.32 ± 1.68	−3.18 ± 1.59	0.209
	J_0_ (D)	0.15 ± 0.34	0.18 ± 0.36	0.098
	J_45_ (D)	0.07 ± 0.27	0.08 ± 0.30	0.541
Hyperopia (*n* = 40)	Sphere (D)	0.86 ± 1.79	2.43 ± 1.54	<0.001
	Cylinder (D)	−0.57 ± 0.96	−0.65 ± 0.96	0.074
	SE (D)	0.58 ± 1.49	2.10 ± 1.18	<0.001
	J_0_ (D)	0.16 ± 0.48	0.22 ± 0.47	0.002
	J_45_ (D)	−0.23 ± 0.23	0.01 ± 0.25	0.005

**Table 2:** Refractive components of both groups measured by VX130 with and without cycloplegia.

Refractive Error	Parameters (Mean ± SD)	Pre-Cycloplegia	Post-Cyclolegia	*p*
Myopia (*n* = 20)	Sphere (D)	−3.54 ± 1.74	−3.37 ± 1.77	0.019
	Cylinder (D)	−0.85 ± 0.82	−0.82 ± 0.79	0.420
	SE (D)	−3.96 ± 1.73	−3.78 ± 1.72	0.012
	J_0_ (D)	0.19 ± 0.41	0.19 ± 0.42	0.967
	J_45_ (D)	0.12 ± 0.36	0.12 ± 0.31	0.905
Hyperopia (*n* = 40)	Sphere (D)	0.82 ± 1.47	1.94 ± 1.57	<0.001
	Cylinder (D)	−0.88 ± 1.02	−0.79 ± 0.87	0.014
	SE (D)	0.37 ± 1.18	1.54 ± 1.27	<0.001
	J_0_ (D)	0.30 ± 0.54	0.28 ± 0.43	0.407
	J_45_ (D)	0.04 ± 0.28	0.09 ± 0.27	0.009

## Data Availability

The datasets used in this study are available on request from the corresponding author.
